# Immune Monitoring of Cancer Patients Prior to and During CTLA-4 or PD-1/PD-L1 Inhibitor Treatment

**DOI:** 10.3390/biomedicines6010026

**Published:** 2018-03-01

**Authors:** Antje Tunger, Maximilian Kießler, Rebekka Wehner, Achim Temme, Friedegund Meier, Michael Bachmann, Marc Schmitz

**Affiliations:** 1 National Center for Tumor Diseases, University Hospital Carl Gustav Carus, TU Dresden, Fetscherstraße 74, 01307 Dresden, Germany; antje.tunger@uniklinikum-dresden.de (A.T.); rebekka.wehner@tu-dresden.de (R.W.); achim.temme@uniklinikum-dresden.de (A.T.); friedegund.meier@uniklinikum-dresden.de (F.M.); m.bachmann@hzdr.de (M.B.); 2 Institute of Immunology, Medical Faculty Carl Gustav Carus, TU Dresden, Fetscherstraße 74, 01307 Dresden, Germany; maximilian.kiessler@tu-dresden.de; 3 Department of Neurosurgery, Section Experimental Neurosurgery and Tumor Immunology, University Hospital Carl Gustav Carus, TU Dresden, Fetscherstraße 74, 01307 Dresden, Germany; 4 Department of Dermatology, University Hospital Carl Gustav Carus, TU Dresden, Fetscherstraße 74, 01307 Dresden, Germany; 5 Department of Radioimmunology, Institute of Radiopharmaceutical Cancer Research, Helmholtz Center Dresden-Rossendorf, Bautzner Landstraße 400, 01328 Dresden, Germany

**Keywords:** cancer immunotherapy, immune monitoring, immune checkpoints, cytotoxic T lymphocyte antigen 4, programmed cell death protein 1, programmed cell death 1 ligand 1

## Abstract

Targeting the immune checkpoint receptors cytotoxic T lymphocyte antigen 4 (CTLA-4), programmed cell death protein 1 (PD-1), or programmed cell death 1 ligand 1 (PD-L1) represents a very attractive treatment modality for tumor patients. The administration of antibodies against these receptors can promote efficient antitumor effects and can induce objective clinical responses in about 20–40% patients with various tumor types, accompanied by improved survival. Based on their therapeutic efficiency, several antibodies have been approved for the treatment of tumor patients. However, many patients do not respond to checkpoint inhibitor therapy. Therefore, the identification of biomarkers is required to guide patient selection for this treatment modality. Here, we summarize recent studies investigating the PD-L1 expression or mutational load of tumor tissues as well as the frequency and phenotype of immune cells in tumor patients prior to and during CTLA-4 or PD-1/PD-L1 inhibitor treatment.

## 1. Introduction

In recent years, immunotherapy of tumors has gained much momentum by the development of novel promising treatment modalities that have been considered as a scientific breakthrough [1]. One of these encouraging strategies is based on the inhibition of immune checkpoint molecules, resulting in improved antitumor responses mediated by CD4^+^ and CD8^+^ T lymphocytes [2,3]. CD8^+^ T cells efficiently recognize and destroy tumor cells. CD4^+^ T cells augment the capacity of dendritic cells (DCs) to induce CD8^+^ T cell responses. They also provide help for the maintenance and expansion of CD8^+^ T cells and can eliminate tumor cells directly. In addition, CD4^+^ T cells are able to promote the differentiation of B cells into antibody-producing plasma cells. The T cell response is initiated through antigen recognition by the T cell receptor (TCR). Furthermore, the amplitude and quality of this response are regulated by the balance between costimulatory and coinhibitory signals [4,5,6]. Under physiological conditions, these inhibitory pathways, so-called immune checkpoints, are crucial for maintaining self-tolerance and thus prevention of autoimmunity as well as for minimizing collateral tissue damage during immune responses against pathogens. Tumors can hijack the immune checkpoint pathways to evade elimination by the immune system. Upregulation of checkpoint molecules is associated with suppression of T cell function, so-called T cell exhaustion. This process leads to the gradual loss of T cell function during chronic viral infections or tumor diseases [7]. Numerous costimulatory and coinhibitory molecules have been identified over the past decades [5,6]. Accordingly, agonist antibodies for costimulatory pathways or antagonist antibodies for inhibitory pathways are in current clinical testing [2,3,8]. Both approaches aim at the amplification and activation of antigen-specific T cell responses, thus enhancing the endogenous antitumor activity.

One prerequisite for understanding how these checkpoint inhibitors promote tumor rejection and for the design of optimized treatment modalities is to monitor the cellular immune response [9,10,11,12]. Immune monitoring technologies help by providing novel insights into the mechanisms underlying checkpoint inhibitor therapy and by identifying potential modes of resistance to treatment. In addition, they enable us to identify biomarkers to distinguish between responders and non-responders and to reduce treatment-related side effects. Here, we review recent studies exploring the PD-L1 expression or mutational load of tumor tissues as well as the frequency and phenotype of tumor-infiltrating or blood-circulating immune cells in tumor patients prior to and during checkpoint inhibitor therapy.

## 2. Cytotoxic T lymphocyte Antigen 4 (CTLA-4/CD152)

### 2.1. Biology of CTLA-4

CTLA-4 is a member of the immunoglobulin superfamily, which is expressed on activated T cells and can function as a negative regulator [13]. On naïve T cells, CTLA-4 is not expressed on the cell surface, but stored within intracellular compartments. Induced by antigen binding to the TCR, CTLA-4 is translocated to the cell surface [14]. Thereby, the extent of CTLA-4 trafficking is dependent on the strength of TCR engagement. CTLA-4 regulates the amplitude of the early T cell response to antigen by binding to CD80 or CD86, thus sharing its ligands with CD28. It outcompetes CD28 for ligand binding by having a much higher affinity for CD80 and CD86 [15]. CTLA-4 functions in contrast to CD28 by dampening T cell activation [15,16]. The exact mechanism by which CTLA-4 suppresses T cell stimulation is still not fully understood. In this context, it has been demonstrated that activation of the protein phosphatases SHP2 and PP2A may play an essential role in counteracting kinase signals that are induced by TCR and CD28 [17]. Furthermore, it has been reported that CTLA-4 increases T cell motility and overrides the TCR-induced stop signal required for immunological synapse formation between T cells and antigen-presenting cells (APCs) [18]. The reduced contact periods between T cells and APCs resulted in decreased T cell proliferation and cytokine production. In addition, binding of CTLA-4 to CD80/86 led to removal of these molecules from the cell surface by trans-endocytosis [19]. Further studies revealed that CTLA-4 enhances the immunosuppressive activity of regulatory T (T_reg_) cells [20,21]. FOXP3 is a transcription factor expressed in T_reg_ cells determining their regulatory phenotype [22]. As CTLA-4 is a target gene of FOXP3, the receptor is constitutively expressed on the surface of T_reg_ cells [20,21]. Considering the physiological role of CTLA-4, the blockade of this molecule in the context of a tumor immunotherapy would be important to directly enhance the functional properties of effector T cells as well as for inhibiting T_reg_ cell-mediated immunosuppression of effector T cells.

The first preclinical report of CTLA-4 blockade using an anti-CTLA-4 antibody was published by Leach and colleagues in 1996, who showed an enhanced immune response leading to an effective tumor elimination [23]. The success of this approach encouraged the testing of anti-CTLA-4 antibodies in clinical trials. Two fully humanized anti-CTLA-4 monoclonal antibodies, ipilimumab and tremelimumab, have been developed. A phase III clinical trial enrolling patients with advanced melanoma demonstrated a significantly improved overall survival (OS) for patients treated with ipilimumab with or without a glycoprotein 100 peptide vaccine compared to the peptide vaccine alone [24]. In a subsequent phase III trial, the combination of the DNA-alkylating agent dacarbazin with ipilimumab showed improved OS in melanoma patients compared to dacarbazin alone [25]. Based on these studies, ipilimumab was approved by the United States Food and Drug Administration (FDA) for the treatment of patients with metastatic melanoma in 2011.

### 2.2. Immune Monitoring

Since ipilimumab is an antibody of IgG1 isotype, which can bind to Fc receptors, it has been postulated that ipilimumab could deplete T_reg_ cells by mediating antibody-dependent cellular cytotoxicity. In this context, it has been shown that ipilimumab treatment depressed T_reg_ cell numbers in the blood of patients [26]. More recently, Romano and colleagues reported that CD16-expressing non-classical monocytes derived from the blood of melanoma patients can essentially contribute to ipilimumab-mediated depletion of T_reg_ cells [27]. However, another clinical trial revealed that anti-CTLA4 antibody-based treatment does not eliminate T_reg_ cells, but expands functional T_reg_ cells [28].

Absolute lymphocyte counts (ALC) > 1000/mm^3^ in peripheral blood at the start of the second course and an increase of the absolute eosinophil counts (AEC) > 100/mm^3^ have been shown to correlate with an improved OS in ipilimumab-treated melanoma patients [29]. In contrast, it has been observed that non-responders to ipilimumab therapy display elevated amounts of neutrophils, monocytes, and monocytic myeloid-derived suppressor cells (MDSCs) at baseline in comparison to responders [30]. More recently, Martens and colleagues reported on a prognostic combination model for ipilimumab-treated melanoma patients comprising several baseline blood biomarkers [31]. They observed that low lactate dehydrogenase (LDH), absolute monocyte counts (AMC), and monocytic MDSCs as well as high AEC, relative lymphocyte counts (RLC), and T_reg_ frequencies were associated with better OS [31]. Furthermore, it has been shown that a high baseline expression of indoleamine 2,3-dioxygenase (IDO) in the tumor tissues is associated with improved clinical outcome of ipilimumab-treated melanoma patients [32].

Further studies investigated the effects of anti-CTLA-4 therapy on the frequency and phenotype of tumor-infiltrating or blood-circulating T cells. Thus, it has been demonstrated that CTLA-4 blockade results in a significant increase of tumor-infiltrating CD8^+^ T cells [33]. Hodi et al. showed that ipilimumab application after vaccination with irradiated, autologous tumor cells engineered to secrete granulocyte-macrophage colony-stimulating factor (GVAX) generates clinical responses in a majority of metastatic melanoma patients [34]. The extent of therapy-induced tumor necrosis was linearly related to the natural logarithm of the ratio of tumor-infiltrating CD8^+^ effector T cells to T_reg_ cells in posttreatment biopsies of metastatic lesions, indicating that ipilimumab can modulate the balance of effector T cells and T_reg_ cells [34]. In addition, upregulation of human leukocyte antigen (HLA)-DR on CD4^+^ and CD8^+^ T cells induced by combined immunotherapy with GVAX and ipilimumab of patients with advanced prostate cancer has also been reported [35]. This effect was greater in patients with a partial response or stable disease than in patients with progressive disease. Furthermore, an upregulation of the activation marker CD40 on blood-circulating CD1c^+^ DCs was observed, which was associated with improved OS [35]. A further study revealed that ipilimumab-treated melanoma patients with New York esophageal squamous cell carcinoma-1 (NY-ESO-1)-specific serum antibodies and CD8^+^ T cells recognizing NY-ESO-1-derived peptides experienced more frequent clinical benefit than those with undetectable CD8^+^ T cell response [36]. Liakou et al. observed that CD4^+^ T cells from peripheral blood and tumor tissues from anti-CTLA-4 antibody-treated bladder cancer patients show increased expression of the costimulatory molecule inducible T cell costimulator (ICOS) [37]. The ICOS^+^ CD4^+^ T cell population contained interferon (IFN)-γ-producing T cells, suggesting that anti-CTLA-4 treatment skewed CD4 effector cells toward a type 1 T helper cell (T_H_1)-like profile [37]. More recently, it has been reported that anti-CTLA-4 therapy induces the expansion of melanoma-infiltrating ICOS^+^ T_H_1-like CD4^+^ T cells as well as exhausted-like CD8^+^ T cells [38]. Furthermore, it has been demonstrated that an increased frequency of ICOS^+^ CD4^+^ T cells, sustained over a period of 12 weeks of anti-CTLA-4 therapy, correlates with improved OS in melanoma patients [39]. In another study, Wang and colleagues observed an upregulation of Ki67, ICOS, and GATA3 in blood CD4^+^ and CD8^+^ T cells of anti-CTLA-4 antibody-treated melanoma patients [40]. In addition, Jacquelot et al. documented that PD-L1 expression on peripheral blood T cells is prognostic on OS and progression-free survival (PFS) in anti-CTLA-4 antibody-treated melanoma patients [41]. When investigating tumor samples from prostate cancer patients prior and after anti-CTLA-4 therapy, Gao et al. detected an increased expression of PD-L1 and V-domain Ig suppressor of T cell activation (VISTA), representing another inhibitory immune checkpoint molecule [42], on CD4^+^ T cells, CD8^+^ T cells, and CD68^+^ macrophages in posttreatment tumor tissues [43]. In further studies, the TCR diversity in peripheral blood of tumor patients treated with anti-CTLA-4 antibodies has been determined. In this context, Cha et al. have shown that CTLA-4 blockade results in an increased TCR diversity and that the maintenance of high-frequency TCR clonotypes during treatment is associated with improved OS [44]. In addition, it has been reported that melanoma patients who experienced clinical benefit from CTLA-4 blockade had a higher degree of richness and evenness in their TCR repertoire than patients who did not have clinical benefit [45]. Hannani et al. found a negative impact of high baseline serum levels of soluble CD25 on the clinical outcome of melanoma patients, indicating that soluble CD25 can predict resistance to CTLA-4 blockade [46].

In another study, the gene expression profiles of tumor biopsies collected from melanoma patients were analyzed before and after ipilimumab treatment. Patients with high expression levels of immune-related genes in tumor biopsies prior treatment were more likely to respond to CTLA-4 blockade [47]. More recently, van Allen and colleagues analyzed whole exomes from pretreatment melanoma biopsies to explore the impact of the mutational and neoantigen load on the response to ipilimumab [48]. They observed that the mutational as well as the neoantigen load in the tumor microenvironment were significantly associated with clinical benefit. These results were confirmed by another study, demonstrating that a significantly improved OS of anti-CTLA-4 antibody-treated melanoma patients was observed when their tumors exhibit a high clonal neoantigen burden [49]. These findings suggest enhanced tumor immunogenicity by increased mutational load through generation of neoantigens, thereby increasing the probability that the patient responds to CTLA-4 blockade. Following such observations, Łuksza and colleagues designed a neoantigen fitness model, which is based on the likelihood of neoantigen presentation by HLA molecules and subsequent T cell recognition, to predict clinical outcome of tumor patients after anti-CTLA-4 therapy [50].

**Figure 1:**
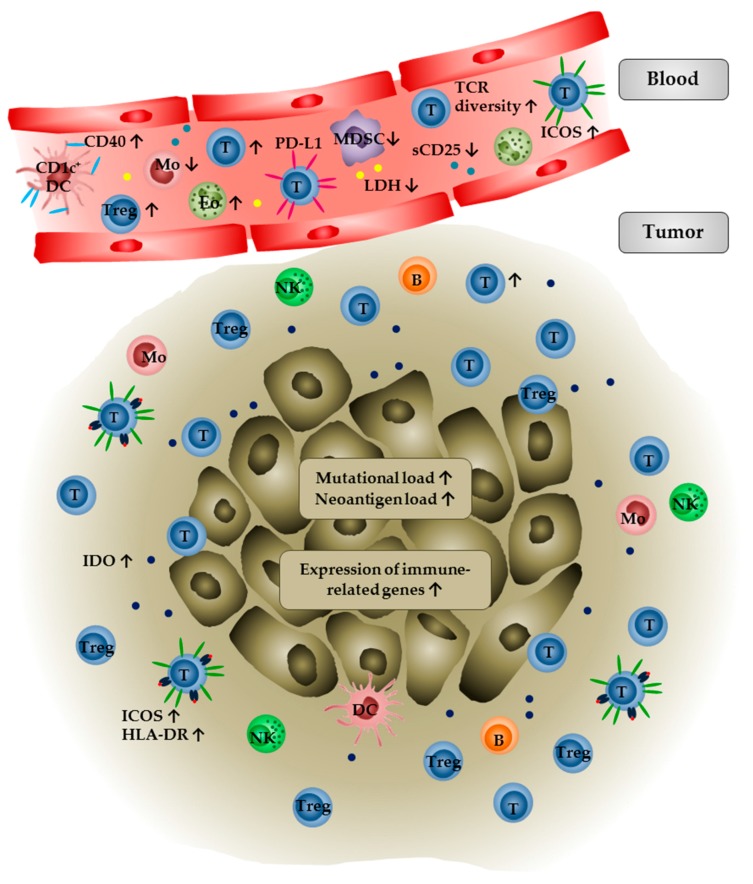
Immunological characteristics in anti-CTLA-4 antibody-treated tumor patients that are associated with clinical outcome. In peripheral blood, higher eosinophil, lymphocyte, and T_reg_ cell numbers, an increased TCR diversity as well as upregulation of CD40 on CD1c^+^ DCs and ICOS on T cells are associated with improved clinical outcome. Furthermore, low numbers of monocytic MDSCs and monocytes as well as low levels of LDH are correlated with clinical benefit. PD-L1 expression on T cells and high levels of soluble CD25 are predictors of resistance to CTLA-4 blockade. Within the tumor, an increased frequency of infiltrating T cells associated with an upregulation of HLA-DR and ICOS as well as increased levels of IDO are correlated with favorable clinical outcome. In addition, a high intratumoral mutational and neoantigen load or a high expression level of immune-related genes increase the probability that the tumor patients respond to anti-CTLA-4 therapy.

A summary of immunological characteristics in tumor or blood samples of anti-CTLA-4 antibody-treated patients that are associated with clinical outcome is shown in Figure 1.

## 3. Programmed Cell Death-1 (PD-1/CD279)

### 3.1. Biology of PD-1

PD-1 is a transmembrane receptor belonging to the immunoglobulin superfamily, which is expressed upon activation on T cells, natural killer (NK) cells, and B cells [5,6]. Therefore, PD-1 blockade may not only enhance T cell activity, but also the lytic function of NK cells as well as antibody production of plasma cells. Furthermore, PD-1 is expressed on T_reg_ cells, enhancing their proliferation after ligand binding [51]. PD-1 binds to PD-L1 and PD-L2. PD-L1 is widely expressed on hematopoietic and non-hematopoietic cells as well as cancer cells and can be induced by proinflammatory cytokines such as IFN-γ. PD-L2 is mainly expressed by APCs and induced mostly by interleukin-4 and granulocyte-macrophage colony-stimulating factor [52,53,54,55]. Interestingly, PD-L1 additionally can bind CD80 on T cells, thereby delivering another inhibitory signal [56]. In contrast to CTLA-4, the major role of PD-1 is not at the initial T cell activation phase, but rather to regulate the immune response of antigen-experienced effector T cells within the peripheral tissues. Activated T cells show an upregulation of PD-1, which persists during their way through the peripheral tissue. The expression of the PD-1 ligands is induced by inflammatory signals in the tissues. Thereby, activation of T cells can be downregulated to prevent the tissue of collateral damage during immune response [52,57]. As a result of PD-1 signaling, proliferation, cytokine production, and cytotoxicity of T cells are impaired and apoptosis is induced [58]. Similarly to CTLA-4, PD-1 engagement leads to inhibition of the TCR-mediated stop signal, which could shorten the duration of the contact between T cells and their target cells [59]. There are multiple ways how PD-1 signaling can modulate T cells, all together targeting on the suppression of T cell immune responses. This pathway is adopted by tumors to prevent themselves from immune attack [60]. These findings provided an important insight for the potential of blocking antibodies for this pathway, as their properties have been shown for chronic viral infection [61]. In different cancer mouse models, an enhancement of the antitumor immunity through antibody blockade of PD-1 or its ligands could be demonstrated [62,63]. In 2010, a first phase I clinical trial using a fully human IgG4 anti-PD-1 antibody in multiple cancer entities was conducted [64]. Treatment was well tolerated and clinical responses were observed in several patients. Further clinical testing of anti-PD-1 therapy revealed objective clinical responses in patients with advanced melanoma [65,66,67], non-small-cell lung cancer (NSCLC) [68,69,70], renal cell carcinoma (RCC) [71,72], bladder cancer [73], and Hodgkin’s lymphoma [74]. Due to these findings, the FDA approved anti-PD-1 antibody treatment for these indications.

### 3.2. Immune Monitoring

Recently, Tumeh et al. investigated the presence of infiltrating CD8^+^ T cells in tissue samples obtained from melanoma patients before and during anti-PD-1 therapy [75]. They found that pretreatment samples from patients who experienced a clinical response show higher densities of CD8^+^ T cells in comparison to samples from patients that progressed during therapy. In addition, they observed that melanoma patients responding to anti-PD-1 therapy display an increase in intratumoral CD8^+^ T cell frequency that was correlated with radiographic reduction of tumor size. Their findings indicate that pre-existing intratumoral CD8^+^ T cells may predict clinical response to anti-PD-1 therapy [75]. More recently, it has been reported that PD-1 blockade predominantly induces expansion of exhausted-like tumor-infiltrating CD8^+^ T cells [38]. Furthermore, Ribas et al. demonstrated that anti-PD-1 antibody treatment resulted in an increased frequency of intratumoral T cells in patients who responded to therapy [76]. The density of tumor-infiltrating B cells and monocytic MDSCs was also increased on treatment. By comparing the immune infiltrates of PD-L1^+^ and PD-L1^−^ melanomas, tumor-infiltrating T-lymphocytes (TILs) displayed an IFN-γ-dominated cytokine expression in PD-L1^+^ melanomas [60]. This can be explained by the mechanism that activated TILs upregulate PD-1 and start IFN-γ secretion after recognition of tumor antigens. As a response, tumor cells increase PD-L1 expression, thereby protecting the tumor from attack of PD1^+^ effector T cells. Based on these findings, PD-L1 expression is considered a marker of an active antitumor immune response. PD-L1 expression by tumor cells and infiltrating immune cells varied significantly by tumor type and was most abundant in melanoma, NSCLC, and RCC [77]. Evidence for an association between intratumoral PD-L1 expression and objective clinical responses in tumor patients treated with anti-PD-1 or anti-PD-L1 antibodies has been demonstrated in various trials [12]. For example, Herbst et al*.* found an association between clinical responses in anti-PD-L1 antibody-treated patients with tumors expressing high levels of PD-L1, especially when PD-L1 was detected on tumor-infiltrating immune cells [78]. Topalian et al*.* reported that 9 of 25 patients with PD-L1^+^ tumors showed an objective response, whereas, out of 17 patients with PD-L1^−^ tumors, none had an objective response [79]. In addition, Garon and colleagues found that PD-L1 expression in at least 50% of tumor cells correlated with improved efficiency of anti-PD-1 therapy in NSCLC patients [68]. These observations indicate that PD-L1 expression may represent a biomarker for clinical response and outcome in trials blocking PD-1/PD-L1 interaction. However, other clinical trials yielded contradictory results [12]. For example, Motzer et al*.* reported that RCC patients with 1% or greater PD-L1 expression have reduced OS compared to patients with less than 1% [72]. Furthermore, Gettinger et al*.* found no clear association between PD-L1 expression and response or survival in anti-PD-1 antibody-treated patients with NSCLC [80].

When investigating a correlation between mutational burden in tumors and sensitivity to PD-1 blockade, Rizvi et al. have shown that a higher nonsynonymous mutation or candidate neoantigen burden in tumors from anti-PD-1-treated NSCLC patients was associated with improved PFS [81]. In line with this observation, Le et al. found that the immune-related objective response rate and immune-related PFS rate in anti-PD-1 antibody-treated patients with mismatch repair-deficient colorectal cancer were higher compared with patients with mismatch repair-proficient colorectal cancer [82]. Whole-exome sequencing revealed a significantly higher number of somatic mutations per tumor in mismatch repair-deficient tumors as compared with mismatch repair-proficient tumors. High numbers of somatic mutations and potential mutation-associated neoantigens were associated with longer PFS [82]. More recently, it has been reported that loss-of-function mutations in the *PBRM1* gene in tumors from anti-PD-1 antibody-treated patients are associated with clinical benefit [83]. When exploring a correlation between intratumoral neoantigen load and sensitivity to PD-1 blockade, McGranahan et al. have reported that a high clonal neoantigen burden in tumors of anti-PD-1 antibody-treated NSCLC patients is associated with improved clinical outcome [49]. In addition, the neoantigen fitness model described by Łuksza et al., which is based on the likelihood of neoantigen presentation by HLA molecules and subsequent T cell recognition, is able to predict clinical outcome of anti-PD-1 antibody-treated tumor patients [50].

To identify potential biomarkers for the prediction of clinical responses, further studies analyzed changes in peripheral blood immune cells and soluble molecules from tumor patients receiving anti-PD-1 antibody treatment. In this context, it has been shown that anti-PD-1 therapy leads to an expansion of PD-1^+^ CD8^+^ T cells in peripheral blood of NSCLC patients [84]. PD-1^+^ CD8^+^ T cell responses were observed in the majority of patients with clinical benefit. A further study revealed that the magnitude of reinvigoration of circulating T cells with an exhausted phenotype determined in relation to pretreatment tumor burden is correlated with clinical responses in anti-PD-1 antibody-treated melanoma patients [85]. In addition to changes in the T cell compartment, Krieg et al. have shown that the frequency of classical blood monocytes at baseline in anti-PD-1 antibody-treated melanoma patients is a predictor of PFS and OS [86]. Furthermore, it has been reported that high relative eosinophil counts, RLC, and low LDH in peripheral blood at baseline are associated with favorable OS of anti-PD-1-treated melanoma patients [87].

Two recent studies have discovered a correlation between the gut microbiome of tumor patients and their clinical response to anti-PD-1 immunotherapy [88,89]. Responding melanoma patients showed a significantly higher alpha diversity and a relative abundance of Ruminococcaceae bacteria in their gut microbiome [88]. In addition, Routy and colleagues found that the relative abundance of *Akkermansia munciniphila* is significantly associated with favorable clinical outcome of patients with advanced cancer [89]. These findings indicate that the gut microbiome markedly influences the efficacy of anti-PD-1 immunotherapy in tumor patients.

**Figure 2:**
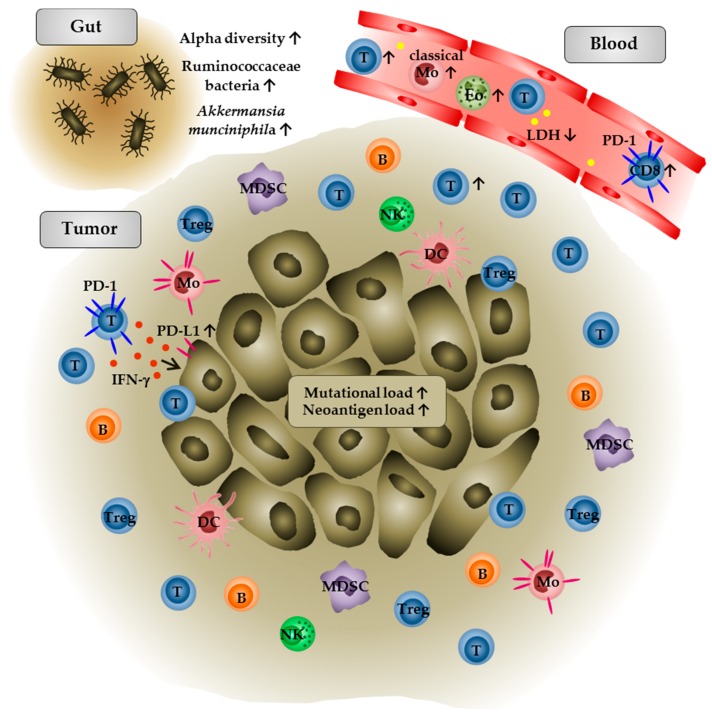
Immunological characteristics in anti-PD-1/PD-L1 antibody-treated tumor patients that are associated with clinical outcome. In peripheral blood, higher numbers of eosinophils, lymphocytes, PD-1^+^ CD8 T cells, and classical monocytes as well as low levels of LDH are associated with improved clinical responses. Within the tumor, higher densities of CD8^+^ T cells in pretreatment tumor samples and an increase in intratumoral CD8^+^ T cell frequencies during anti-PD-1 therapy are detectable in patients that show a clinical response. Further studies indicate that a high PD-L1 expression on tumor cells and infiltrating immune cells as well as a high intratumoral mutational and neoantigen load are correlated with an improved survival of patients. In addition, a significantly higher alpha diversity or a relative abundance of Ruminococcaceae bacteria and *Akkermansia munciniphila* in the gut microbiome are associated with favorable clinical outcome of tumor patients.

A summary of immunological characteristics in tumor or blood samples of anti-PD-1/PD-L1 antibody-treated patients that are associated with clinical outcome is given in Figure 2.

## 4. Conclusions

Immunotherapeutic strategies targeting CTLA-4 or the PD-1/PD-L1 axis induce objective clinical responses and improve survival in patients with various tumor types, including melanoma, NSCLC, and RCC. However, many patients do not respond to anti-CTLA-4 or anti-PD1/PD-L1 therapy. Therefore, the identification of biomarkers to select patients responding to treatment and for monitoring the course of disease to adapt therapy is needed. Immune monitoring technologies exploring tumor cells and tumor-infiltrating or blood-circulating immune cells in patients are useful to define biomarkers as predictors of clinical response and to reduce treatment-related side effects. For example, it has been demonstrated that an increased frequency of blood-circulating ICOS^+^ CD4^+^ T cells, sustained over a period of 12 weeks of anti-CTLA-4 therapy, correlates with favorable clinical outcome in melanoma patients. Further studies revealed that PD-L1 expression on tumor cells and infiltrating immune cells is associated with objective clinical responses in anti-PD-1 antibody-treated patients and may therefore represent a biomarker for clinical outcome. However, other clinical trials yielded contradictory results. There are some potential factors contributing to the varying reported outcomes when investigating an association between PD-L1 expression and clinical responses in different patient cohorts, such as diverse cancer types or cancer stages. Various assays and antibodies as well as variable cut-off values and different scoring methods are currently used to define PD-L1^+^ cells by immunohistochemistry. Therefore, the standardization of methods is needed to obtain comparable results from different studies. Immune monitoring is also useful to identify potential modes of resistance to immune checkpoint inhibitor therapy. Thus, a significantly higher expression of PD-L1 and VISTA on T cells, CD8^+^ T cells, and CD68^+^ macrophages in tumor tissues from prostate cancer patients after anti-CTLA-4 therapy was reported. The expression of the inhibitory immune checkpoints PD-L1 and VISTA may explain the poor responsiveness of prostate cancer patients to CTLA-4 blockade and supports the design of a combination therapy targeting both molecules.
